# The EU one-stop-shop collection of publicly available information on COVID-19 *in vitro* diagnostic medical devices

**DOI:** 10.12688/f1000research.27308.1

**Published:** 2020-11-03

**Authors:** Mauro Petrillo, Maddalena Querci, Olga Tkachenko, Ioana-Raluca Siska, Enrico Ben, Alexandre Angers-Loustau, Alessia Bogni, Antonino Brunetto, Marco Fabbri, Linda Garlant, Antoon Lievens, Amalia Munoz, Valentina Paracchini, Danilo Pietretti, Antonio Puertas-Gallardo, Barbara Raffael, Eleonora Sarno, Virginie Tregoat, Fabrizio Zaro, Guy Van den Eede

**Affiliations:** 1 European Commission, Joint Research Centre (JRC), Ispra, Italy; 2 European Commission, Directorate General for Health and Food Safety (SANTE), Brussels, Belgium; 3 Past affiliation: European Commission, Joint Research Centre (JRC), Ispra, Italy; 4 Current affiliation: European Commission, European Publication Office, Luxembourg, Luxembourg; 5 GFT Italia s.r.l., Milan, Italy; 6 European Commission, Joint Research Centre (JRC), Geel, Belgium; 7 Past affiliation (until 30-06-2020): European Commission, Joint Research Centre (JRC), Ispra, Italy; 8 Engineering Ingegneria Informatica S.p.A, Taino, Italy

**Keywords:** In-vitro diagnostics, covid-19, sars-cov-2, detection method

## Abstract

The *JRC COVID-19 In Vitro Diagnostic Devices and Test Methods Database*, aimed to collect in a single place all publicly available information on performance of CE-marked *in vitro* diagnostic medical devices (IVDs) as well as *in house* laboratory-developed devices and related test methods for COVID-19, is here presented. The database, manually curated and regularly updated, has been developed as a follow-up to the Communication from the European Commission “Guidelines on *in vitro* diagnostic tests and their performance” of 15 April 2020 and is freely accessible at https://covid-19-diagnostics.jrc.ec.europa.eu/.

## Introduction

The Communication from the Commission “Guidelines on *in vitro* diagnostic tests and their performance” ^1^, published on 15 April 2020, states the following under the *Further Actions Needed* section: “ *The Commission, supported by the ECDC, health technology assessment experts and in vitro diagnostics competent authorities, will assist Member States with a centralised overview of available information on test performance and act as a single point of contact for management of this information. Taking stock of the state of the art on a regular basis will support Member States’ informed decisions on national testing strategies, as well as support the continuous development of devices by manufacturers.*”

As an initial step in collecting performance information of devices and *in house* methods, to address the above need, European Commission services (Directorate-General for Health and Food Safety [DG SANTE], Directorate-General Joint Research Centre [DG JRC], Directorate-General for Research and Innovation [DG RTD]), together with the European Centre for Disease Prevention and Control (ECDC), several experts from *in vitro* diagnostics competent authorities and from the European Network for Health Technology Assessment (EUnetHTA) ^1^, published the working document "Current performance of COVID-19 test methods and devices and proposed performance criteria" ^2^ on 16 April 2020.

The JRC capitalised on its expertise in knowledge management to conduct the literature review as part of this work and, as a follow-up action to the need identified in the Communication, committed to make the information broadly accessible and to update the compilation as new data become available.

The outcome of these actions is the **JRC COVID-19 *In Vitro* Diagnostic Devices and Test Methods Database** presented here, a single place collection of all publicly available information on performance of CE-marked *in vitro* diagnostic medical devices (IVDs) as well as *in house* laboratory-developed devices and related test methods for COVID-19. The database is freely accessible at https://covid-19-diagnostics.jrc.ec.europa.eu/.

## Methods

### Information retrieval

The initial information is gathered following the strategy for documentation on test methods and devices indicated in Section 3 "Methodology used" of the *Current performance of COVID-19 test methods and devices and proposed performance criteria - Working document of Commission services* ^2^.

### Verification of information

The retrieved information is manually verified and curated. In particular:

•    For CE-marked devices, only the information that the manufacturer has chosen to make publicly available is included in the database. Full information on the manufacturer’s performance evaluation of the device is contained in the technical documentation required by EU legislation ^3^. As manufacturer technical documentation is usually not publicly available and is therefore not included, a form is available to stakeholders to update and integrate the information: manufacturers are invited to submit performance information on new devices, which are not yet listed in the database, or to provide data not available to the authors at the time of the last update. The submitted information, once verified against the source provider, is taken into consideration for updating the database.

•    Performance details (as retrieved from manufacturers’ web pages) are provided only for devices commercially available with CE-IVD mark. Products labelled as for ‘research use only’ or ‘under development’ as well as products fulfilling regulatory frameworks other than the one in place in the EU are listed for information only. The correctness of information, such as performance data of the listed devices, has not been confirmed by checking raw experimental data or full technical documentation of the manufacturer nor by own laboratory verification or by any clinical validation studies.

•    A team of JRC experts regularly takes care of updating the Scientific Literature section, which includes scientific articles reporting about the use and performances of devices and related test methods. This section regards also the so-called *in house* ^2^ or laboratory developed devices, which are used in healthcare institutions but are not commercially available. These are not included in the list of devices, as they are not easily identifiable by name, but scientific publications on such devices and related test methods are included in the section.

•    Information on publicly available results of validation studies and on publications by national/regional health technology assessment bodies and Joint Action EUnetHTA ^4^ is included. These reports are produced in response to requests from national authorities and support national policy and decision-making on testing for COVID-19.

### In silico NAAT methods simulations

*In silico* simulations of NAAT methods, on available high-quality and full length sequenced SARS-CoV-2 genomes (provided as collaboration with GISAID ^5^) are performed by using ThermonucleotideBLAST ^6^. The tool is run on each assembled genome with default parameters, except for the following ones: *-e=30, -E=20, --max-gap=4, --max-mismatch=4*. Putative not redundant target amplicons are then extracted to build the final target dataset that is added to the database.

At the time of writing, high-quality (i.e. <1% Ns and <0.05% unique amino acid mutations) and full length (i.e. >29,000 bp) sequenced SARS-CoV-2 genomes have been manually downloaded from the GISAID website. To simplify the sharing of information and the related analysis, a formal agreement is under finalisation between GISAID and JRC. 

## Results

The database is structured in a way aimed to:

•    facilitate sharing of information among researchers, in line with the principle of FAIR sharing;

•    link the devices, and their features, to scientific articles that reported their use and performance, in order to have a clear, transparent and fully open data source where users can look at and make the right choice in the selection of the device or method to use;

•    verify and monitor if natural occurring SARS-CoV-2 mutations give rise to "false negative".

Data records are currently organised and available in three main sections:

1. **COVID-19 *in vitro* diagnostic medical devices -** This section gives access to publicly available *in vitro* diagnostic medical devices for COVID-19 and is being updated periodically. Additional performance (as retrieved from manufacturers’ web pages) is provided only for devices or kits commercially available with CE-IVD mark.
  Direct link to this dataset page, where data can be downloaded in CSV format, is: https://covid-19-diagnostics.jrc.ec.europa.eu/devices.
2. **Scientific literature on COVID-19 test methods and devices** - This section allows browsing on performance of test methods and devices for COVID-19 diagnostics retrieved from selected scientific articles and is being updated periodically.
  Direct link to this dataset page, where data can be downloaded in CSV format, is: https://covid-19-diagnostics.jrc.ec.europa.eu/literature.
3. **SARS-CoV-2 target regions -** This section is an inventory of PCR-based nucleic acid amplification tests (NAATs) used by laboratories and *in silico* simulations of these assays on available high-quality and full length sequenced SARS-CoV-2 genomes (in collaboration with GISAID ^5^). The latter are pre-computed values corresponding to the extent of matching of the primers and probes from NAAT database methods against high-quality, full length genomic sequences, which are made available by GISAID to enable this analysis.

In addition, the database has pointers to the EUnetHTA publications repository. EUnetHTA Joint Action 3 is the scientific and technical component of EU cooperation on HTA. It builds on years of long standing collaboration between HTA agencies, financially supported by the EU since early 2000. The current Joint Action (2016 – 2021) is co-funded by the EU Health Programme and includes government appointed organisations and a large number of relevant regional agencies and not-for-profit organisations that produce or contribute to HTA in Europe (i.e. 86 organisations from 26 Member States plus Norway, Serbia, Switzerland, Ukraine and United Kingdom). Responding to the needs of policy makers and public health authorities for researched, timely and reliable information, EUnetHTA has launched its COVID-19-related repository of publications and outputs. The repository gathers publications by EUnetHTA and by HTA organisations on testing methods and devices, but also on treatment options, and other public health measures relevant to COVID-19. Direct link to this page is: https://covid-19-diagnostics.jrc.ec.europa.eu/eunethta.

Finally, a **Submit your device** section provides forms for manufacturers and NAAT developers to submit information on devices not yet listed in the database or to provide performance data not available to the authors at the time of the last update. The submitted information, once verified against the source provider, is taken into consideration for updating the database.

**Figure 1.**
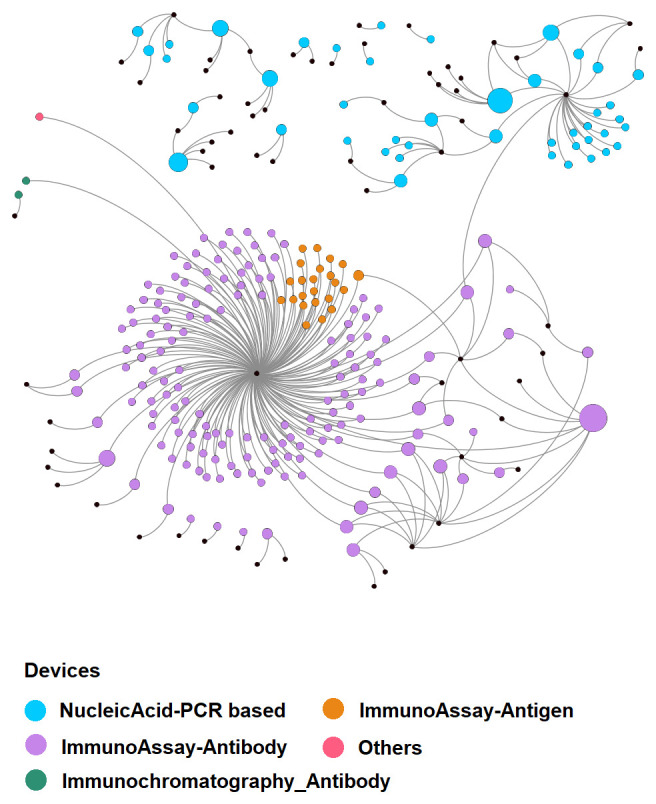
Device-article relations. This graph visualises the relations (grey lines) among devices (coloured according to the corresponding detection principle) and the scientific articles (black spots) in which they are mentioned. The large “corona” near the centre and the group near the top right corner are clustered around two documents that refer to 172 ^9^ and 23 ^10^ devices, respectively. It is quite evident that publications are specialised with respect to devices detection principle (i.e. the used technology): few publications (only three) mention different detection principles' devices. Please note that devices without references are not represented. The size of the symbol representing the device is proportional to the corresponding number of references (min 1, max 8).

At the time of writing, the database includes 869 devices and 382 selected articles, easily linked to each other, as shown in Figure 1. Considering the rapidly evolving situation in relation to the development and commercialisation of diagnostic devices for COVID-19, the completeness of the information is limited to the time of the last update as indicated in the database for each individual item. For this reason, manufacturers are invited to submit and update the information through the corresponding "Submit your device" section's form.

**Figure 2.**
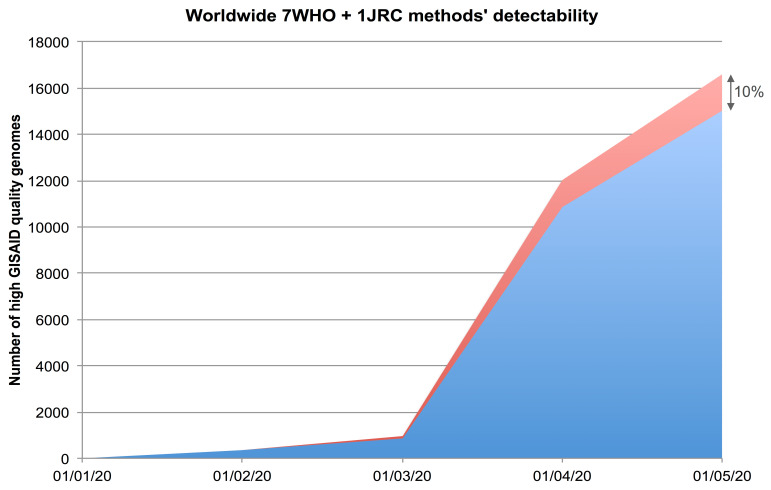
NAAT methods target detectability. As of 01 May 2020, more than 16,000 high quality (<1% Ns and < 0.05% unique amino acid mutations) and full length (> 29,000 bp) viral genomes were made available by GISAID ^5^. By performing *in silico* PCR simulations with eight NAAT methods, the graph shows that about 10% of the 16,000 selected genomes (cumulative numbers) were found to be not detectable *in silico* by at least one of the eight NAAT methods. Blue area represents the (cumulative) number of genomes *in silico* detected by all eight NAAT methods; red area represents the (cumulative) number of those not detected by at least one NAAT method.

**Figure 3.**
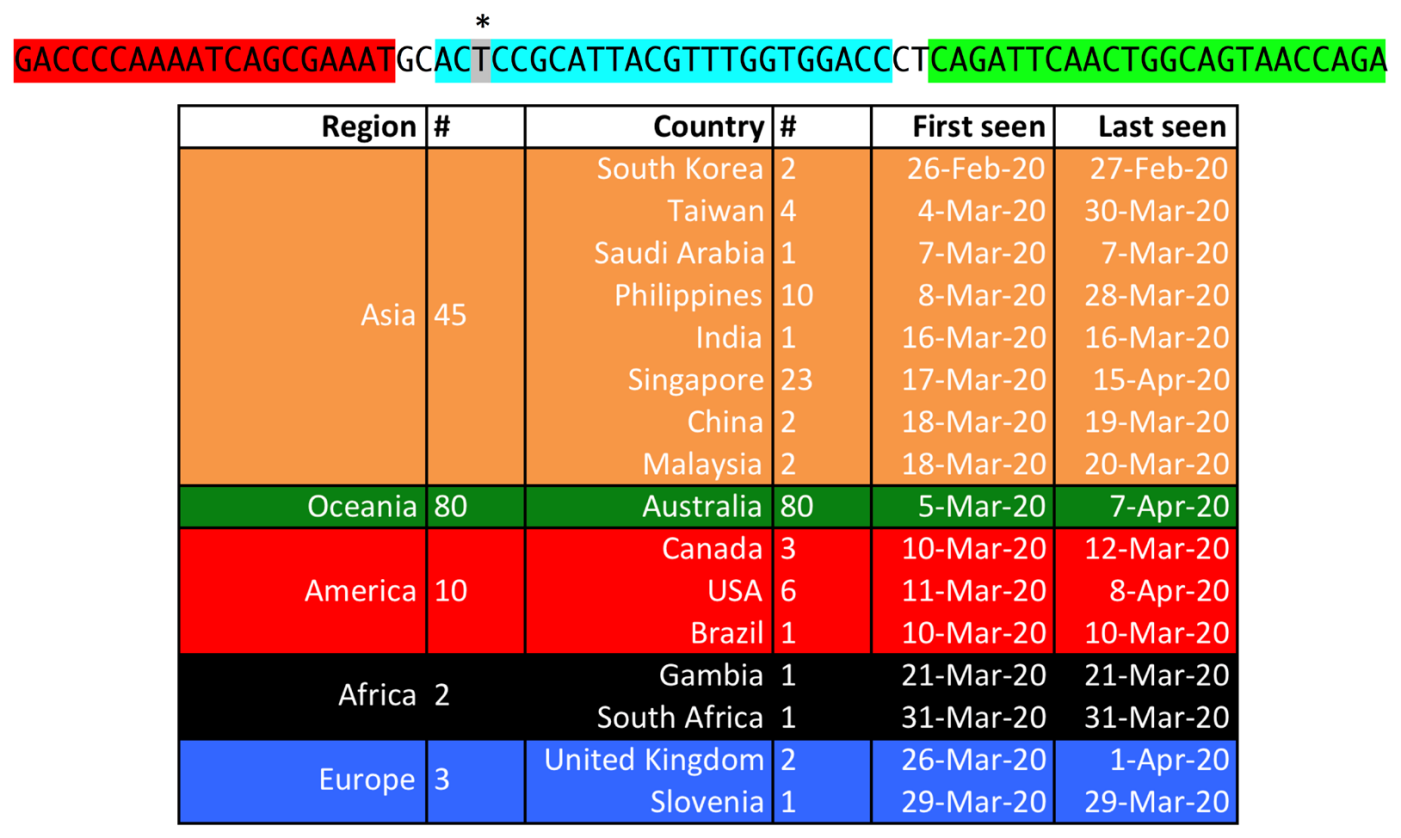
Genomic variations that can affect NAAT methods target detectability. As of 01 May 2020, more than 16,000 high quality (<1% Ns and < 0.05% unique amino acid mutations) and full length (> 29,000 bp) viral genomes were made available by GISAID ^5^. An example of identified genomic variations that can affect NAAT methods target detectability is here reported and it regards the first method developed by the US Centers for Disease Control and Prevention and supported by WHO in 7. 1) On the top, the target sequence (in red the left primer, in green right primer, and in azure the probe annealing region) that has been found in several genomes. With respect to the expected reference target, a variation (C/T transition, marked by *) is present at the beginning of the probe annealing region that can potentially affect the detection method performance. 2) Below, the table with details about the genomes where this variation of the target sequence has been found: the two columns with # indicate the number of genomes with such a sequence, aggregated by region and country, respectively. In the table, the dates of first and last appearance of the variation are reported for each country, in order to provide information on the "spread" of the variation.

With respect to NAAT methods, currently eight NAAT methods ** are available in the database: seven are from the World Health Organization (WHO) support to COVID-19 and are *in-house* PCR protocols assays posted online on the WHO website ^7^ while one was developed by the JRC in the context of the production of EURM-019, a universal positive control material to be used in the testing of SARS-CoV-2 by RT-PCR (described in reference 8). The possibility to evaluate whether variations occurring overtime in the viral genomes can affect PCR-based detection methods is fundamental to guarantee reliability of the detection. The aims of the **SARS-CoV-2 target regions** section in the database are therefore both to detect potential target regions of the NAAT methods and to highlight possible differences with the expected reference sequence that might affect the performance of NAATs. As of 01 May 2020, about 10% of the retrieved genomes were found to be not detectable *in silico* by at least one of the eight NAAT methods, as shown in Figure 2. As an example, a case of variation potentially affecting NAAT methods detectability is shown in Figure 3.

As for devices, considering the rapidly evolving situation in relation to COVID-19, NAAT methods developers are invited to submit updated information or new developed ones through the corresponding "Submit your device" section's form.

## Discussion

According to our knowledge, it is the first time that a repository provides and links information on COVID-19 *in vitro* diagnostic medical devices and the scientific articles testing and reporting their performance.

To date, more than 100 requests, either for addition of new *in vitro* diagnostic medical devices or for updating the information, have been received through the forms complied by manufacturers, as demonstration of the strong need of having a structured data sharing point for this kind of information. However, it is important to highlight that this resource does NOT represent a list of devices approved or authorised for use either by the European Commission or by Member States’ national authorities. There is no central approval system for *in vitro* diagnostic medical devices in the EU. The currently applicable legislation for placing COVID-19 diagnostic devices on the market in the EU is Directive 98/79/EC on *in vitro* diagnostic medical devices ^3^. Under the current Directive ^3^, for COVID-19 devices that are designed for professional use, the manufacturer may affix the CE-mark to the product after having ensured the compliance of the device with the Directive and drawn up a declaration of conformity. For COVID-19 devices that are designed for use by lay persons (self-tests), the manufacturer must also apply to a third party body called a notified body that will do additional verification and issue a certificate. As a consequence, the JRC should not be deemed responsible for the validity of such data.

As recently reported by Guglielmi ^12^, “ *even once a test is working beautifully in the lab, it still faces an arduous journey to mass usage. The first challenge is to verify performance, because quality can vary*”. We believe that the here presented database contributes to the required prompt handling of the on-going public health COVID-19 situation. It makes sharing information easier and helps medical professionals, scientists and laboratories understand and properly assess COVID-19 medical devices quickly. The overview of the market may also help manufacturers develop and improve their own devices, in line with the current EU legislation.

## Data availability

The database is freely accessible at https://covid-19-diagnostics.jrc.ec.europa.eu/. CSVs of the ‘COVID-19 *in vitro* diagnostic medical devices’ and ‘Scientific literature on COVID-19 test methods and devices’ databases at the time of publication have been archived on Zenodo repository (see below). However, the database is updated very frequently, and the latest data can be downloaded in these sections (described in Results) as CSVs.

Zenodo: JRC COVID-19 In Vitro Diagnostic Devices and Test Methods Database. http://doi.org/10.5281/zenodo.4117601 ^13^.

This project contains the following underlying data:

- covid-19-methods.csv (CSV of COVID-19 *in vitro* diagnostic medical devices database at time of publication)
- covid-19-literature.csv (CSV of Scientific literature on COVID-19 test methods and devices database at time of publication)

Data are available under the terms of the Creative Commons Attribution 4.0 International license (CC-BY 4.0).

The ‘SARS-Cov2 target regions’ database is enabled using third party data from GISAID, subject to GISAID’s Terms and Conditions. The database is freely accessible at https://covid-19-diagnostics.jrc.ec.europa.eu/amplicons and GISAID data are available directly from GISAID at https://www.gisaid.org/ ^5^. Access to GISAID data requires registration and agreement to the conditions for use at https://www.gisaid.org/registration/register/.

## Code availability

Code used to perform scientific literature mining is freely available at https://github.com/ec-jrc/JRC_COV19_IVD-DEVs-TEMs_DB.

Archived code at time of publication: https://doi.org/10.5281/zenodo.4084600 ^14^.

License: BSD-3-Clause
