# Supplementary material for: The EU one-stop-shop collection of publicly available information on COVID-19 in vitro diagnostic medical devices
Source: F1000Res. 2020 Nov 3;9:1296. [Version 1] doi: 10.12688/f1000research.27308.1 (PMC7851715; doi:10.12688/f1000research.27308.1)
Supplement: Peer review history

# Peer review: Reviewer response for version 1

**DOI:** 10.5256/f1000research.30175.r75227

**Published:** 2021-02-01

**Authors:** Christopher Viljoen

**Affiliations:** 1 Human Molecular Biology Unit, School of Biomedical Sciences, University of the Free State, Bloemfontein, South Africa

- The Abstract would be strengthened with an introductory sentence regarding context to COVID-19 and the need for such information.
- The Introduction needs a short contextualized introduction to the need for this type of information in a COVID-19 pandemic.
- The Introduction could also indicate how the Commission has responded to the need for the information as indicated above.
- The paragraph on COVID-19 mutations and test methods needs to include some context to the problem.
- The Discussion - 1st paragraph - should indicate "in vitro diagnostic medical devices and assays".
- The article would benefit from some concluding comments reiterating the importance of having access to the information presented in the database.

Are sufficient details of methods and materials provided to allow replication by others?

Yes

Is the rationale for creating the dataset(s) clearly described?

Partly

Are the datasets clearly presented in a useable and accessible format?

Yes

Are the protocols appropriate and is the work technically sound?

Yes

Reviewer Expertise:

Human Molecular Biology

I confirm that I have read this submission and believe that I have an appropriate level of expertise to confirm that it is of an acceptable scientific standard.

# Peer review: Reviewer response for version 1

**DOI:** 10.5256/f1000research.30175.r75984

**Published:** 2021-01-06

**Authors:** Angelo Facchiano

**Affiliations:** 1 Institute of Food Science, National Research Council, Avellino, Italy

The manuscript presents a significant work aimed to collect publicly available information on test methods for COVID-19. Due to the amount of devices and methods available, the database described offers a useful tool to be addressed in the field. I find of special interest the example on genomic variations that can affect detectability.

 The manuscript is well presented and written.

 I find in the manuscript only minor points to be addressed:

- As a  reader, I would find in the article an example of the output obtained when the database is searched.
- I would not find explanation of abbreviations in the abstract if not really used there.
- abbreviations should be explained at the first appearance (check for NAAT; IVD - if removed from abstract, FAIR - explain or add a reference; and so on).
- under keywords, check upper/lower case.

Are sufficient details of methods and materials provided to allow replication by others?

Yes

Is the rationale for creating the dataset(s) clearly described?

Yes

Are the datasets clearly presented in a useable and accessible format?

Yes

Are the protocols appropriate and is the work technically sound?

Yes

Reviewer Expertise:

Bioinformatics, biochemistry, molecular biology

I confirm that I have read this submission and believe that I have an appropriate level of expertise to confirm that it is of an acceptable scientific standard.
